# Fatherhood alters gene expression within the MPOA

**DOI:** 10.1093/eep/dvy026

**Published:** 2018-12-12

**Authors:** Adele M H Seelke, Jessica M Bond, Trent C Simmons, Nikhil Joshi, Matthew L Settles, Danielle Stolzenberg, Mijke Rhemtulla, Karen L Bales

**Affiliations:** 1 Department of Psychology, University of California, Davis, Davis, USA; 2 Bioinformatics Core Facility, University of California, Davis, Davis, USA; 3 California National Primate Research Center, University of California, Davis, Davis, USA

**Keywords:** fathering, dendritic spines, plasticity, gene expression, RNA sequencing, parenting

## Abstract

Female parenting is obligate in mammals, but fathering behavior among mammals is rare. Only 3–5% of mammalian species exhibit biparental care, including humans, and mechanisms of fathering behavior remain sparsely studied. However, in species where it does exist, paternal care is often crucial to the survivorship of offspring. The present study is the first to identify new gene targets linked to the experience of fathering behavior in a biparental species using RNA sequencing. In order to determine the pattern of gene expression within the medial preoptic area that is specifically associated with fathering behavior, we identified genes in male prairie voles (*Microtus ochrogaster)* that experienced one of three social conditions: virgin males, pair bonded males, and males with fathering experience. A list of genes exhibiting different expression patterns in each comparison (i.e. Virgin vs Paired, Virgin vs Fathers, and Paired vs Fathers) was evaluated using the gene ontology enrichment analysis, and Kyoto Encyclopedia of Genes and Genomes pathways analysis to reveal metabolic pathways associated with specific genes. Using these tools, we generated a filtered list of genes that exhibited altered patterns of expression in voles with different amounts of social experience. Finally, we used NanoString to quantify differences in the expression of these selected genes. These genes are involved in a variety of processes, with enrichment in genes associated with immune function, metabolism, synaptic plasticity, and the remodeling of dendritic spines. The identification of these genes and processes will lead to novel insights into the biological basis of fathering behavior.

## Introduction

Biparental care, in which both mother and father contribute to the care of the offspring, is displayed by a minority of mammalian species – usually cited as 3–5% [1–3]. Female parenting is obligate because mammalian offspring need to nurse, but the participation of the male is seen only in our own and a limited number of other mammalian species [4–8]. In species where paternal care does exist, including humans, it is often crucial to the survivorship of offspring; or at least has significant long-term impacts on growth as well as neural, reproductive, and social development [9, 10]. However, much is still unknown about the specific hormonal and neurobiological regulation of paternal care [6].

The vast majority of parenting research focuses on the mother, while the role of the father has mostly been considered in the context of paternal absence [9, 11–13]. Considering paternal care through the absence of the father in a biparental species has drawbacks, however, since it confounds the quantitative absence of another caregiving individual with the qualitative absence of the father in particular. Paternal absence is the most extreme situation. Individual variation in fathering can also have long-term effects on offspring [9], and in the context of nonhuman mammals is always carried out in a biparental care situation. In prairie voles, we have shown that natural variation in biparental parenting behavior predicts pup development and juvenile social behavior [14], exploratory behavior and pair-bonding, and adult aggression and stress responses [15–17]. It is not always possible in an intact biparental family to disentangle which outcomes in offspring are due to maternal care and which are due to paternal care. However, some very interesting roles for the father have been observed. For instance, males may compensate for poor maternal care (or allow mothers to expend less energy on non-nutritive tasks like carrying) [14, 18]; or a paternal behavior such as retrievals (carrying pups back to the nest or territory) may be directly linked to offspring display of retrievals and aggression as an adult [19, 20].

The hormonal mechanisms underlying fathering behavior have been much less studied than those underlying maternal behavior, although it has been hypothesized that similar neural circuits are responsible for both maternal and paternal behaviors [21]. While alterations in neural activity with parenthood appear to be hormonally regulated in females, hormonal manipulation in males has often resulted in outcomes that are either ambiguous or species-specific [22]. For instance, testosterone is inversely related to paternal care in most species [9, 23, 24], but is obligate for paternal care in California mice [25]. Prolactin, another leading candidate for the regulation of male parenting, decreases male parenting when administered, as well as when blocked [26]. These inconsistencies have led some to suggest that across species, paternal behavior depends upon nonhomologous neuroendocrine circuits [6], and has raised the question of what factors are involved in the generation of these behaviors.

Although the hormonal regulation of parenting may vary by sex, it is believed that the neural circuit governing parental behavior is similar in mothers and fathers [27]. The medial preoptic area of the hypothalamus (MPOA) is a central node in the neural circuit that regulates both maternal and paternal care and has long been recognized as playing a critical role in the generation and regulation of parental behavior (see [9, 28] for reviews). In biparental California mice, paternal experience increases Fos immunoreactivity in the MPOA [29]. Virgin male prairie voles that were exposed to pups also showed an increase in Fos immunoreactivity within the MPOA [30]. Lesions of the MPOA disrupt both maternal and paternal behavior in California mice [31]. In California mouse males, aromatase levels within the MPOA vary in response to parental status [32], while another study in male California mice showed a decrease in progesterone receptor mRNA expression in the MPOA of fathers compared with virgin males [33]. Studies in mice, which are not parental in the wild but can show induced paternal care in the laboratory [34–36], have bolstered the view of a central role for the MPOA in paternal care.

The goal of this study was to identify novel gene targets and potential mechanisms that may contribute to the production and regulation of paternal behavior. We analyzed gene expression in three groups of adult male prairie voles: virgin males, males who had formed a pair bond with a female, and males who had both pair bonded and gained fathering experience. Samples were taken from the MPOA, a region that is central to the expression of both maternal and paternal behaviors [21, 37–39], and RNA was extracted and sequenced.

## Materials and Methods

### Subjects

Subjects were 18 adult male prairie voles. Animals were born and housed in the Psychology Department Vivarium at the University of California, Davis. These animals were descendants of a wild stock originally caught near Champaign, IL. The animals were weaned at 20 days of age and pair housed with an animal of the same sex (sibling if available, similarly aged nonsibling if not) in small laboratory cages (27 × 16 × 13 cm) in which food and water were available *ad libitum.* All animals were maintained at ∼70°F (21°C) on a 14:10 light/dark cycle with the lights on at 6 a.m. All experiments were performed under National Institutes of Health guidelines for the care of animals in research and were approved by the Institutional Animal Care and Use Committee of the University of California, Davis.

At postnatal day (P) 42–45 subjects were placed in one of three groups of age-matched males: (i) virgin males, (ii) sexually experienced, ‘pair-bonded’ males, or (iii) males with fathering experience. This was designed to dissociate alterations in gene expression that were related to pair bonding from alterations related to paternal behavior. Virgin males were housed with a male same-age conspecific for ∼20 days, and they were euthanized without engaging in sexual contact with females. Pair-bonded males were housed with a same-age female conspecific for ∼20 days, after which the males were euthanized. Because mating and pregnancy strengthens pair bonds in prairie voles [40–43], we confirmed that females were pregnant. Pair-bonded males were euthanized before females gave birth, ensuring they had no contact with pups. The third group consisted of males that had 3 days of paternal experience. These males were also housed with female pair-mates with whom they presumably formed a pair-bond. The females gave birth, and the males were permitted three days of contact with pups before they were euthanized. Three days of parental experience was chosen to minimize age differences between subjects. Furthermore, prairie vole fathers already exhibit large amount of paternal care by postnatal day 3 [14, 44].

**Figure 1:**
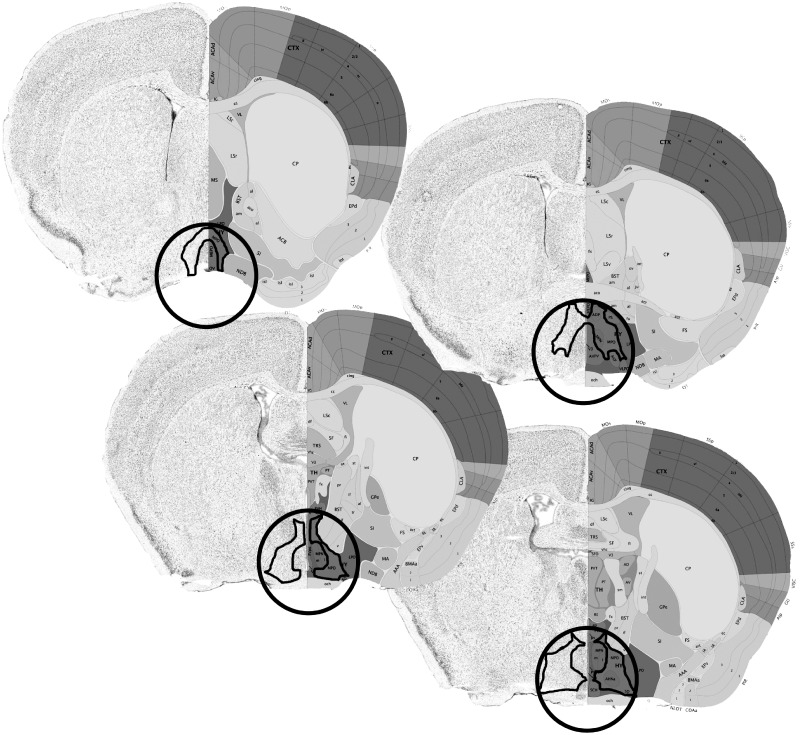
A schematic representing the area from which tissue samples were taken. The circumference of the tissue punch is delineated by a circle, and the MPOA is outlined in black. The tissue punches removed the entirety of the MPOA, as well as small portions of adjacent hypothalamic tissue

Subjects were anesthetized using isoflurane and euthanized via cervical dislocation. Upon euthanasia, brains were removed and flash frozen. The brains were sliced on a cryostat into 120 µm sections and mounted on slides. Punches were taken from the MPOA using a 15.5-gauge blunt needle (Fig. 1) and were stored in a −80°C freezer until RNA extraction.

### RNA Extraction

Total RNA was isolated with Qiazol reagent (Qiagen, Valencia, CA) and purified with an RNeasy^®^ Plus Micro Kit (74004; Qiagen) as well as the optional DNase digestion (Qiagen 129046). A Nanodrop™ Spectrophotometer was used to determine the quality and the quantity of the RNA. All samples had a 260/280 ratio >1.8.

### RNA Sequencing

A total of 18 RNA-seq libraries were prepared from the RNA of the 18 male prairie voles (Table 1). RNA sequencing and library preparation was performed by the DNA Technologies and Expression Analysis Core in the Genome Center of the University of California, Davis. A total RNA analysis ng sensitivity (Eukaryotes) of all 18 samples resulted in a mean RIN of 9.2 (range 8.3–9.9). Barcoded RNA-seq libraries were generated from 1 μg total RNA each after poly-A enrichment using the Kapa Stranded RNA-seq kit (Kapa Biosystems, Cape Town, South Africa) following the instructions of the manufacturer. The libraries were generated on a Sciclone G3 liquid handler (Caliper Life Sciences, Alameda, CA). Quality was verified with the Bioanalyzer 2100 instrument (Agilent, Santa Clara, CA) and quantified by fluorometry on a Qubit instrument (Life Technologies, Carlsbad, CA) and pooled in equimolar ratios. The pooled library was then quantified by qPCR with a Kapa Library Quant kit (Kapa Biosystems) and sequenced on one lane of an Illumina HiSeq 4000 (Illumina, San Diego, CA) with paired-end 150 bp reads.

**Table 1:** RNA data

ID	Raw reads	Trimmed reads	% Reads kept	% Aligned	% Aligned to rRNA
V1	25764898	25657648	99.58373598	95.1	5.67
V2	20747099	20665472	99.60656186	95.99	2.63
V3	23633483	23500072	99.43550005	96.13	2.07
V4	17827384	17709907	99.34103063	96.06	2.38
V5	22459377	22360940	99.56171091	95.59	4.09
V6	22690769	22591612	99.56300732	96.09	2.78
P1	22451193	22317681	99.40532336	92.7	8.72
P2	23931482	23837973	99.60926365	95.84	2.26
P3	21258757	21155922	99.51626993	94.97	5.00
P4	20114070	20011415	99.48963586	95.86	3.16
P5	21770575	21677246	99.57130668	96.11	2.06
P6	19282593	19185302	99.49544649	95.73	3.28
F1	20833672	20702987	99.3727222	95.14	4.82
F2	21680207	21568888	99.48654088	95.99	2.53
F3	20565896	20479195	99.57842342	96.06	1.65
F4	19759347	19680046	99.59866589	96.17	2.27
F5	21472445	21367346	99.51054014	95.95	3.06
F6	22055401	21961628	99.57482977	96.31	2.32

Raw sequencing data have been deposited at NCBI’s sequence read archive under study accession number SRP128134.

### Bioinformatic Analysis

Bioinformatic analysis was performed by the UC Davis Bioinformatics Core Facility, also in the Genome Center. Briefly, reads were trimmed for adapter contamination and quality using scythe (version c128b19) and sickle (version 7667f147e6), respectively. The reads were then aligned to the prairie vole genome (MicOch1.0) using bwa mem (version 0.7.13), after which featureCounts (version 1.5.0-p1) was used to create the raw gene expressions counts. Finally, R (version 3.3.2) with the edgeR and limma/voom packages were used to filter and transform (voom transformation), and test for statistical significances between groups. Briefly, data were prepared by first choosing to keep genes that achieved at least 0.5 count per million in at least five samples, normalization factors were calculated using trimmed mean of *M*-value, and the voom transformation was applied. A completely randomized design was implemented, comparisons of interest were extracted using contrasts, and moderated statistics were computed using the empirical Bayes procedure eBayes. Finally, each gene was corrected for multiple testing using the Benjamini–Hochberg false discovery rate correction. Gene expression was directly compared between each pair of groups, resulting in three comparisons: Virgin males vs Paired males (V vs P), Virgin males vs Fathers (V vs F), and Paired males vs Fathers (P vs F). No genes reached a statistical significant threshold (adjusted *P*-value <0.05) in any of the three pairwise comparisons.

### Gene Ontology Analysis

In order to capture the genes that were most likely to show common functional differentiation between comparison groups we performed gene set enrichment analysis with Gene Ontologies (cellular component, molecular function, or biological process). Enrichment testing was conducted using the Kolmogorov–Smirnov test as implemented in the Bioconductor package topGO [45]. We next examined the gene ontology (GO) annotations that were significantly enriched (*P*-value < 0.05) and filtered the GO annotations in each comparison that were related to the brain or behavior, excluding unrelated annotations (i.e. GO: 0003014, Renal system process or GO: 0008354, Germ cell migration). We then categorized the remaining annotations based on gross function within each comparison group.

### Kyoto Encyclopedia of Genes and Genomes Pathways Analysis

Kyoto Encyclopedia of Genes and Genomes (KEGG) pathways offer insight into how genes interact within biological and metabolic processes. We identified individual genes associated with significantly enriched GO annotations and ran each individual gene through the KEGG pathways database (http://www.genome.jp/kegg/) to identify molecular signaling pathways associated with that gene. A single gene may be involved in a number of different pathways, so we then identified commonly recurring pathways associated with the individual genes. Pathways that were unrelated to brain function (e.g. those that were involved in kidney, liver, or heart metabolism) were not included in the analysis. When a specific gene was associated with multiple pathways of interest it was identified as a candidate gene for further analysis. For example, *Grin2a* was associated with six pathways that are involved in neural plasticity.

We identified 49 candidate genes across 9 biologically significant KEGG pathways and examined how their expression changed between conditions of social experience. For each gene, we standardized expression relative to virgin males. This allowed us to identify whether there were coordinated changes between genes that were involved in a specific pathway. We averaged gene expression across animals within each condition and transformed the data into ratios; the values we used for all analyses were the ratios of gene expression in each condition relative to the virgin condition. For each KEGG pathway, we determined the mean relative expression for all genes in each condition. The expression ratio of genes in virgin animals was set at 1, a value >1 indicated that genes were more expressed relative to virgins, and a value <1 indicated that genes were less expressed relative to virgins. Thus, we analyzed whether gene expression for each gene varied between paired animals and fathers. Effect size was measured using Cohen’s *d.*

### Assessment of Gene Interaction Networks

After generating a list of candidate genes, we analyzed the connectivity of the gene network using the STRING Database (string-db.org) [46]. The STRING database identifies protein–protein interactions between members of a gene set, which allows the user to build a network of functional gene interactions. STRING also measures the functional and interaction enrichments of the gene network, calling upon GO annotations, KEGG pathways, and connections between nodes.

### NanoString Analysis

Following the identification of candidate genes, we performed a quantitative analysis of the expression of 33 genes (30 target genes and 3 housekeeping genes) using the nCounter SPRINT profiler (NanoString Technologies, Seattle, WA). Genes were chosen to be included in the NanoString analysis based on their log fold change values as determined by the expression data, as well as their functional significance. One additional gene, *Bdnf*, was chosen due to previous studies indicating that it plays a significant role in plasticity and parenting [47, 48]. The nCounter analysis assay was conducted using RNA that remained after the completion of the sequencing experiment.

Briefly, NanoString is a medium-throughput method that can analyze many genes within a single sample with comparable sensitivity and accuracy to quantitative real-time RT-PCR [49]. NanoString designed and manufactured custom probes corresponding to the 33 genes we identified for quantitative analysis, consisting of 30 target genes and 3 housekeeping genes (*Gusb*, *Pgk1*, and *Eif4a2*). A code set specific to a 100-base region of the target mRNA was designed using a 3’ biotinylated capture probe and a 5’ reporter probe tagged with a specific fluorescent barcode. Data were collected using the nCounter Digital Analyzer by counting the number of individual barcodes.

Each transcript of interest was recognized by a capture probe and a reporter probe, each containing 30–50 bases complementary to the target mRNA. To minimize assay variability, the code sets also included negative and positive control reporter probes that were developed by the External RNA Control Consortium (ERCC). Six positive control reporter probes (ERCC-selected mRNA targets) were pre-mixed with (Spike-Ins) the code set at a concentration range (0.125–128 fM), a range corresponding to the expression levels of most mRNA of interest, to control for overall efficiency of probe hybridization and determine the detection range for transcripts of interest in each assay. A scaling factor was calculated for each sample, and a scaling factor outside the range of 0.3–3 indicated suboptimal hybridization. In our samples, the scaling factor always fell within the optimal range and was thus applied to all counts in the sample.

Quantitative expression data from the nCounter were downloaded and analyzed using the nSolver software package (NanoString Technologies). The raw counts for all transcripts were multiplied by the scaling factor to produce the adjusted counts. The relative expression was determined for each comparison group, and the effect size of the difference between expression values was determined using Cohen’s *d.* Expression was also compared using *t*-tests, and *P*-values were adjusted for multiple comparisons in nSolver.

## Results

### GO Analysis

Individual genes are associated with GO annotations in order to describe the various functions of a particular gene product. The cellular component analysis describes the locations of gene expression, at the levels of subcellular structures. The molecular function analysis describes the function that each gene product performs within the cell. The biological process analysis describes a recognized series of events or collection of molecular functions associated with a gene or gene product. Each analysis was completed for all genes with differential patterns of expression between the three comparison groups, *V* vs *P*, *V* vs *F*, and *P* vs *F.* Because each GO annotation references many genes, in some instances the same GO annotation was present in multiple comparison groups.

**Figure 2:**
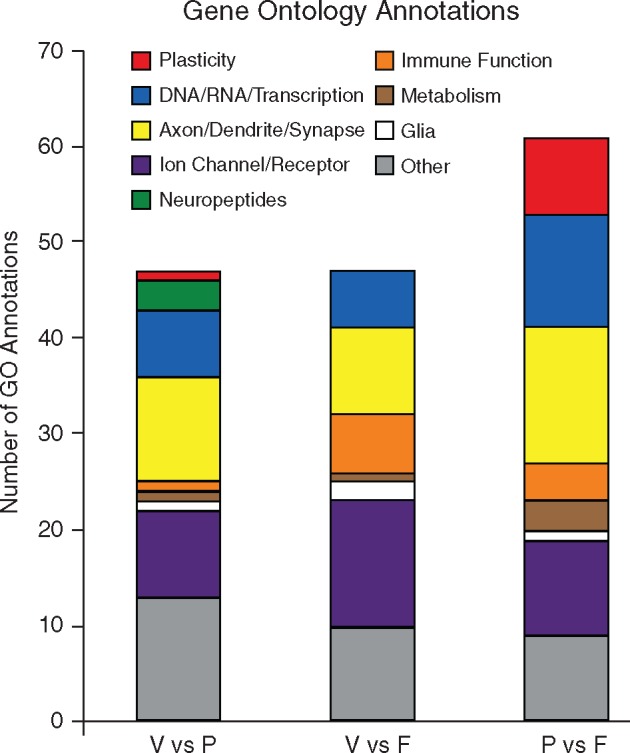
enrichment of GO annotations across comparison groups. The gene enrichment analysis grouped the differentially expressed genes using GO annotations data. We selected significantly enriched GO annotations and identified the annotations that were involved in brain or behavioral processes. Those annotations were then categorized by function within each comparison group. We identified nine functional groups: plasticity (red), DNA/RNA/Transcription (blue), axon/dendrite/synapse (yellow), ion channel/receptor (purple), neuropeptides (green), immune function (orange), metabolism (brown), glia (white), and other (gray). We saw differences in the relative distribution of GO annotation functional groups across the comparison groups. Neuropeptides were only seen in the *V* vs *P* group, whereas the *V* vs *F* group showed a high number of annotations related to immune function. The *P* vs *F* group contained the largest number of annotations related to plasticity, DNA/RNA/transcription, and axon/dendrite/synapse

The initial GO enrichment analysis returned 209 GO annotations in the *V* vs *P* comparison, 222 annotations in the *V* vs *F* comparison, and 264 annotations in the *P* vs *F* comparison that were significantly enriched. Upon selecting the GO annotations in each comparison that were related to the brain or behavior, we were left with 47 GO annotations in the *V* vs *P* comparison, 47 annotations in the *V* vs *F* comparison, and 61 annotations in the *P* vs *F* comparison (Tables 2–4). We then categorized these annotations based on gross function (Fig. 2). The functional categories of GO annotations were differentially distributed across the three comparison groups. Annotations related to Neuropeptide activity were only found in the *V* vs *P* comparison, whereas immune function annotations were most predominant in the *V* vs *F* comparison. The P vs F comparison contained the greatest number of annotations related to Plasticity, DNA/RNA/Transcription, and Axon/Dendrite/Synapse.

**Table 2:** virgin vs paired GO annotations

GO ID	GO Annotation	# genes	Raw *P* value
GO: 0032286	Central nervous system myelin maintenance	4	0.003
GO: 0044224	Juxtaparanode region of axon	6	0.0034
GO: 0019933	cAMP-mediated signaling	61	0.0056
GO: 0045597	Positive regulation of cell differentiation	475	0.006
GO: 0050790	Regulation of catalytic activity	800	0.0063
GO: 0048406	Nerve growth factor binding	4	0.0076
GO: 0035749	Myelin sheath adaxonal region	4	0.0078
GO: 0042043	Neurexin family protein binding	5	0.008
GO: 0008277	Regulation of G-protein coupled receptor protein signaling pathway	53	0.0081
GO: 0061002	Negative regulation of dendritic spine morphogenesis	4	0.0083
GO: 0007218	Neuropeptide signaling pathway	16	0.0092
GO: 0071277	Cellular response to calcium ion	28	0.0106
GO: 0042102	Positive regulation of T-cell proliferation	38	0.0117
GO: 0098656	Anion transmembrane transport	58	0.012
GO: 0042048	Olfactory behavior	6	0.0129
GO: 0044548	S100 protein binding	9	0.0132
GO: 0043679	Axon terminus	29	0.0138
GO: 0048485	Sympathetic nervous system development	13	0.014
GO: 0061014	Positive regulation of mRNA catabolic process	24	0.0145
GO: 0006401	RNA catabolic process	107	0.0147
GO: 0005184	Neuropeptide hormone activity	5	0.0152
GO: 0043220	Schmidt–Lanterman incisure	8	0.0158
GO: 0002052	Positive regulation of neuroblast proliferation	12	0.0158
GO: 1902711	GABA-A receptor complex	4	0.016
GO: 1900271	Regulation of long-term synaptic potentiation	11	0.0168
GO: 0051965	Positive regulation of synapse assembly	49	0.0176
GO: 2000144	Positive regulation of DNA-templated transcription, initiation	10	0.018
GO: 0022851	GABA-gated chloride ion channel activity	3	0.018
GO: 0000123	histone acetyltransferase complex	56	0.0186
GO: 0035976	Transcription factor AP-1 complex	5	0.019
GO: 0008626	Granzyme-mediated apoptotic signaling pathway	3	0.0194
GO: 0021879	Forebrain neuron differentiation	38	0.0198
GO: 0048011	Neurotrophin TRK receptor signaling pathway	15	0.0202
GO: 2000147	Positive regulation of cell motility	235	0.0212
GO: 0071933	Arp2/3 complex binding	4	0.0216
GO: 0005125	Cytokine activity	15	0.0217
GO: 0007271	Synaptic transmission, cholinergic	7	0.0228
GO: 0035176	Social behavior	31	0.0249
GO: 0035198	miRNA binding	11	0.0255
GO: 0070723	Response to cholesterol	10	0.0256
GO: 0005856	Cytoskeleton	919	0.0257
GO: 0005272	Sodium channel activity	23	0.0263
GO: 0042391	Regulation of membrane potential	194	0.0276
GO: 0050775	Positive regulation of dendrite morphogenesis	13	0.0283
GO: 0008188	Neuropeptide receptor activity	15	0.0299
GO: 0006814	Sodium ion transport	69	0.0316
GO: 0008021	Synaptic vesicle	52	0.0355

**Table 3:** virgin vs father GO annotations

GO ID	GO annotation	# genes	Raw *P* value
GO: 0001975	Response to amphetamine	11	0.00033
GO: 0016310	Phosphorylation	981	0.00097
GO: 0007191	Adenylate cyclase-activating dopamine receptor signaling pathway	6	0.00172
GO: 1903861	Positive regulation of dendrite extension	16	0.00206
GO: 0043278	Response to morphine	7	0.00239
GO: 0060391	Positive regulation of SMAD protein signal transduction	8	0.00258
GO: 0005254	Chloride channel activity	29	0.0027
GO: 0000082	G1/S transition of mitotic cell cycle	95	0.00378
GO: 0005516	Calmodulin binding	36	0.0038
GO: 0042110	T-cell activation	210	0.00406
GO: 0008091	Spectrin	3	0.006
GO: 0043406	Positive regulation of MAP kinase activity	98	0.00781
GO: 0071277	Cellular response to calcium ion	28	0.0082
GO: 0019228	Neuronal action potential	12	0.0093
GO: 0097440	Apical dendrite	4	0.0098
GO: 0030857	Negative regulation of epithelial cell differentiation	23	0.01001
GO: 0051098	Regulation of binding	214	0.01046
GO: 0007626	Locomotory behavior	143	0.01116
GO: 0014002	Astrocyte development	18	0.01447
GO: 0010862	Positive regulation of pathway-restricted SMAD protein phosphorylation	20	0.0151
GO: 0007249	I-kappaB kinase/NF-kappaB signaling	104	0.01513
GO: 0048681	Negative regulation of axon regeneration	9	0.01835
GO: 0005815	Microtubule organizing center	413	0.0185
GO: 0048715	Negative regulation of oligodendrocyte differentiation	10	0.0188
GO: 0001963	Synaptic transmission, dopaminergic	19	0.01883
GO: 0001726	Ruffle	80	0.0191
GO: 0008023	Transcription elongation factor complex	29	0.0194
GO: 0019233	Sensory perception of pain	38	0.0198
GO: 0060158	Phospholipase C-activating dopamine receptor signaling pathway	4	0.02016
GO: 0043235	Receptor complex	178	0.0212
GO: 0002407	Dendritic cell chemotaxis	6	0.02176
GO: 0005912	Adherens junction	151	0.0224
GO: 0019538	Protein metabolic process	2271	0.02608
GO: 0017146	NMDA selective glutamate receptor complex	8	0.0274
GO: 0042098	T-cell proliferation	88	0.03093
GO: 0031340	Positive regulation of vesicle fusion	4	0.03095
GO: 0003909	DNA ligase activity	3	0.0332
GO: 0002682	Regulation of immune system process	531	0.03451
GO: 0005921	Gap junction	9	0.0375
GO: 0000778	Condensed nuclear chromosome kinetochore	4	0.0399
GO: 0071144	Heteromeric SMAD protein complex	2	0.0451
GO: 0035240	Dopamine binding	4	0.0455
GO: 0099604	Ligand-gated calcium channel activity	19	0.0469
GO: 0032444	Activin responsive factor complex	2	0.0471
GO: 0005247	Voltage-gated chloride channel activity	2	0.0475
GO: 0001591	Dopamine neurotransmitter receptor activity, coupled via Gi/Go	2	0.0479
GO: 0001042	RNA polymerase I core binding	2	0.0493

**Table 4:** paired vs father GO annotations

GO ID	GO Annotation	# genes	Raw *P* value
GO: 0005955	Calcineurin complex	4	0.00104
GO: 0050840	Extracellular matrix binding	33	0.00172
GO: 0046959	Habituation	4	0.0031
GO: 0007626	Locomotory behavior	144	0.0032
GO: 2001223	Negative regulation of neuron migration	7	0.004
GO: 0046330	Positive regulation of JNK cascade	64	0.0043
GO: 0060079	Excitatory postsynaptic potential	36	0.0043
GO: 0060391	Positive regulation of SMAD protein signal transduction	8	0.0047
GO: 0015116	Sulfate transmembrane transporter activity	3	0.00473
GO: 0070723	Response to cholesterol	10	0.0048
GO: 0001696	Gastric acid secretion	6	0.005
GO: 0051281	Positive regulation of release of sequestered calcium ion into cytosol	20	0.005
GO: 0060395	SMAD protein signal transduction	38	0.0051
GO: 0042755	Eating behavior	10	0.0057
GO: 0033192	Calmodulin-dependent protein phosphatase activity	4	0.00583
GO: 0000403	Y-form DNA binding	4	0.00644
GO: 0010001	Glial cell differentiation	118	0.0065
GO: 0048407	Platelet-derived growth factor binding	11	0.0071
GO: 0017134	Fibroblast growth factor binding	11	0.00772
GO: 0016575	Histone deacetylation	32	0.008
GO: 0045893	Positive regulation of transcription, DNA-templated	783	0.009
GO: 0005516	Calmodulin binding	36	0.01032
GO: 0007616	Long-term memory	18	0.0104
GO: 0005882	Intermediate filament	30	0.01192
GO: 0061014	Positive regulation of mRNA catabolic process	24	0.0122
GO: 0060080	Inhibitory postsynaptic potential	9	0.0124
GO: 0035418	Protein localization to synapse	22	0.0141
GO: 0008009	Chemokine activity	4	0.01592
GO: 0007015	Actin filament organization	169	0.0166
GO: 0070410	Co-SMAD binding	8	0.01861
GO: 0007212	Dopamine receptor signaling pathway	20	0.0198
GO: 0001973	Adenosine receptor signaling pathway	5	0.0199
GO: 0005102	Signaling receptor binding	554	0.02035
GO: 0000978	RNA polymerase II proximal promoter sequence-specific DNA binding	232	0.02098
GO: 0005736	DNA-directed RNA polymerase I complex	7	0.02166
GO: 0004930	G-protein coupled receptor activity	110	0.02186
GO: 0050882	Voluntary musculoskeletal movement	6	0.0224
GO: 0000307	Cyclin-dependent protein kinase holoenzyme complex	27	0.02343
GO: 0030374	Ligand-dependent nuclear receptor transcription coactivator activity	24	0.02383
GO: 0097110	Scaffold protein binding	35	0.02393
GO: 0008622	Epsilon DNA polymerase complex	3	0.02522
GO: 0005881	Cytoplasmic microtubule	29	0.02579
GO: 0000118	Histone deacetylase complex	28	0.02583
GO: 0099061	Integral component of postsynaptic density membrane	2	0.02667
GO: 0044309	Neuron spine	40	0.02738
GO: 0044295	Axonal growth cone	7	0.02809
GO: 0071144	Heteromeric SMAD protein complex	2	0.02824
GO: 0043197	Dendritic spine	37	0.02853
GO: 0043235	Receptor complex	179	0.0291
GO: 0015271	Outward rectifier potassium channel activity	5	0.0317
GO: 0042805	Actinin binding	16	0.03185
GO: 0003700	DNA binding transcription factor activity	385	0.03189
GO: 0019905	Syntaxin binding	25	0.03526
GO: 0098831	Presynaptic active zone cytoplasmic component	2	0.03658
GO: 0005794	Golgi apparatus	562	0.04007
GO: 0000976	Transcription regulatory region sequence-specific DNA binding	368	0.04263
GO: 0017016	Ras GTPase binding	161	0.04338
GO: 0030864	cortical actin cytoskeleton	34	0.04449
GO: 0014069	Postsynaptic density	77	0.04529
GO: 0060053	Neurofilament cytoskeleton	2	0.04637
GO: 0008076	Voltage-gated potassium channel complex	41	0.04825

### KEGG Pathways Analysis

In the *V* vs *P* comparison group, the commonly recurring pathways included: protein export, protein processing in the endoplasmic reticulum, thyroid hormone synthesis, antigen processing and presentation, Ras signaling, Rap1 signaling, neuroactive ligand–receptor pathway, calcium signaling, and regulation of the actin cytoskeleton. In the *V* vs *F* comparison group, the commonly recurring pathways included: protein processing in the endoplasmic reticulum, regulation of the actin cytoskeleton, Ras signaling, metabolic pathways, axon guidance, protein processing in the endoplasmic reticulum, thyroid hormone synthesis, and antigen processing and presentation. In the *P* vs *F* comparison group, the commonly recurring pathways included: Ras signaling, Rap1 signaling, neuroactive ligand–receptor pathway, calcium signaling, MAPK signaling, LTP, glutamatergic pathways, dopaminergic pathways, and regulation of the actin cytoskeleton.

**Figure 3:**
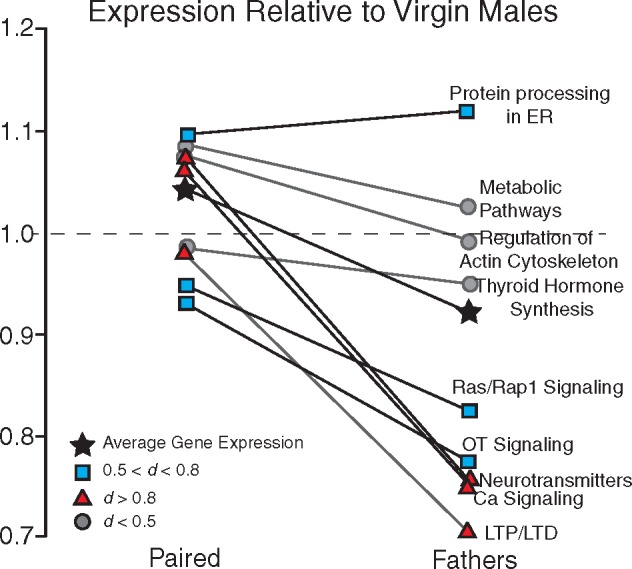
gene expression in paired males and fathers relative to virgin males. Using the KEGG pathways analysis, we identified genes with differential patterns of expression that were linked to nine pathways of biological or behavioral significance. The mean expression of genes associated with each pathway in fathers was averaged and compared against expression in virgin males. On the whole, gene expression was decreased in fathers relative to both virgins and paired males, suggesting that patterns of gene expression undergo coordinated changes in expression relative to social experience. Of the nine pathways, only one showed an increase in gene expression in fathers (protein processing in the endoplasmic reticulum), while five showed decreases in gene expression in fathers (Ras/Rap1 signaling, oxytocin signaling, neurotransmitters, calcium signaling, and LTP/LTD). The overall average gene expression is indicated by black stars. Values that exhibited large effect sizes (Cohen’s *d* > 0.8) are indicated by red triangles, values that exhibited medium effect sizes (0.5 < Cohen’s *d* < 0.8) are indicated by blue squares, and values that exhibited small effect sizes (Cohen’s *d* < 0.5) are indicated by gray circles

Using the list of genes generated from the GO annotations analysis, we next identified genes that were associated with multiple KEGG pathways. By excluding genes that were not associated with any KEGG pathways, or were associated with pathways that were not related to brain function, we further narrowed the range of genes of interest. Ultimately, in each comparison group we identified genes with patterns of expression that differed across social experience and that were linked to biological pathways within the brain (Table 5). We standardized the expression of each gene relative to its expression in virgin males then grouped genes that were associated with nine commonly recurring KEGG pathways and compared the expression of those genes across groups. Since this was an exploratory study, we did not perform statistical tests, and instead used Cohen’s *d* as a measure of effect size (Table 6). Figure 3 shows the changes in gene expression in paired males (left) and fathers (right) compared with virgin males (dashed line). Overall, we observed only moderate changes in gene expression in paired males, but fathers exhibited an overall decrease in gene expression, especially in genes that were associated with long-term potentiation (LTP) and long-term depression (LTD) (*d* = 1.072), neurotransmitters (*d* = 0.911), and Ca^2+^ signaling (*d* = 0.877). We found medium effects of differential patterns of expression in genes that were associated with oxytocin signaling (*d* = 0.787), protein processing in the endoplasmic reticulum (*d* = 0.599), and Ras/Rap1 signaling (0.578). These results suggest that the genes associated with these KEGG pathways undergo coordinated changes in expression patterns that are related to social experience. Furthermore, different social experiences can result in dramatically different patterns of gene expression, i.e. Ca^2+^ signaling.

**Table 5:** genes with altered patterns of expression

Comparison	Genes
V vs P	Cckar, Dnajc3, Enah, Hspa5, Hyou1, Pak3, Pdia3, Pdia4, Rala, Sorbs1, Th
V vs F	Arpc5, Baiap2, Cbl, Chrm1, Chrna1, Cyp2s1, Derl1, Dnajc3, Elovl1, Elovl6, Enah, Epha2, Erp29, Faah, Glra3, Hspa5, Itgb4, Kcnj4, Ksr1, Lamtor3, Nf2, Pdia3, Pdia4, Pdia6, Pigh, Pigo, Pla2g16, Pomgnt2, Prkgc, Pvrl3, Rdx, Tram1, Txndc5
P vs F	Adcy4, Adora2a, Atp2b1, Baiap2, Cacna2d3, Cacnb3, Cckbr, Chrm1, Ddn, Dlg4, Gabrd, Gpr156, Grin2a, Grin2b, Ifngr1, Itpr1, Kcnj2, Kcnj4, Kcnn3, Kdr, Lama2, Ngef, P2rx3, Park2, Prkcg, Ptk2b, Rasgrf2, Rgs14, Rin1, Sipa1l1, Tiam1

**Table 6:** KEGG pathways associated with differentially expressed genes

Neurotransmitters	Adcy4, Adora2a, Cacna2d3, Cacnb3, Cckar, Cckbr, Chrm1, Dlg4, Gabrd, Gpr156, Grin2a, Grin2b, Itpr1, Kcnj2, Kcnj4, P2rx3, Prkcg, Rin1
Ca signaling	Adcy4, Adora2a, Cckar, Cckbr, Chrm1, Grin2a, Grin2b, Itpr1, P2rx3, Ptk2b, Rin1
OT signaling	Adcy4, Cacna2d3, Cacnb3, Kcnj2, Kcnj4, Itpr1, Prkcg
Regulation of actin cytoskeleton	Arpc5, Baiap2, Chrm1, Enah, Itgb4, Rdx, Tiam1, Enah, Pak3
Ras/Rap1 signaling	Adcy4, Adora2a, Epha2, Grin2a, Grin2b, Kdr, Ksr1, Pak3, Pla2g16, Prkgc, Rala, Rasgrf2, Rgs14, Rin1, Sipa1l1, Tiam1
Protein processing in ER	Derl1, Dnajc3, Erp29, Hspa5, Hyou1, Pdia3, Pdia4, Pdia6, Tram1, Txndc5
Thyroid hormone synthesis	Adcy4, Itpr1, Prkcg, Pdia4, Hspa5
LTP/LTD	Grin2a, Grin2b, Itpr1, Rin1

### STRING Database Analysis

We used the STRING database to assess the network connectivity between the genes in each comparison group that were identified as having both differential patterns of expression and functional significance.

**Figure 4:**
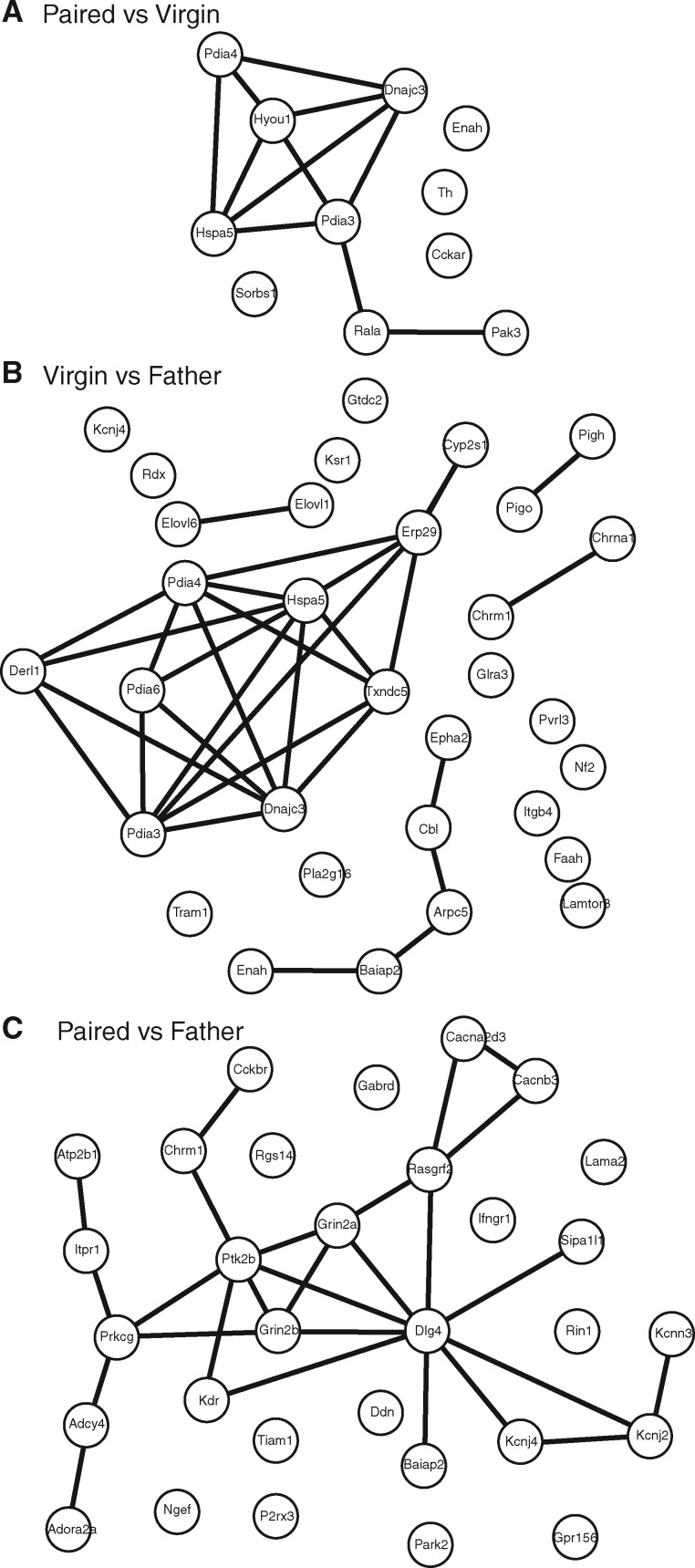
STRING database analysis of gene product interaction networks. Selected genes were run through the STRING database of gene product interactions, and networks were generated for each comparison. (**A**) Paired vs Virgin network; (**B**) Virgin vs Father; (**C**) Paired vs Father

The 11 genes from the *V* vs *P* comparison group produced a network with 11 nodes and 11 edges, and a protein-protein interaction (PPI) enrichment *P*-value of 5.86 × 10^−7^ (Fig. 4A). Thus, the proteins expressed by these genes have significantly more interactions than would be expected by chance, as defined as a random set of similarly sized proteins selected from the genome. There was one cluster of seven interacting proteins, and the functions of these gene products were primarily related to functions of the endoplasmic reticulum, as well as the cellular response to stimulation.

The 33 genes from the *V* vs *F* comparison group produced a network with 32 nodes and 29 edges, and a PPI enrichment *P*-value of 6.99 × 10^−15^ (Fig. 4B), indicating that the proteins expressed by these genes have significantly more interactions than would be expected by chance. These gene products produced one large cluster of nine interacting proteins, one medium cluster of five interacting proteins, and three separate small clusters of two interacting proteins. The large cluster was predominantly involved with the function of the endoplasmic reticulum. The medium cluster was involved with process of neural plasticity, including signaling pathways and modification of the actin cytoskeleton. The three small clusters were involved with the elongation of fatty acid chains, the formation of cholinergic receptors, and GPI-anchor synthesis.

The 31 genes from the *P* vs *F* comparison group produced a network with 31 nodes and 27 edges, and a PPI enrichment *P*-value of 1.36 × 10^−12^ (Fig. 4C), indicating that the proteins expressed by these genes have significantly more interactions than would be expected by chance. These gene products produced 1 large network consisting of 20 interacting proteins. The genes in this network were involved in a variety of functions, including synaptic plasticity and neural transmission, ion transmembrane transport, the cellular response to stimulus, and the structure of the synapse and dendrite.

### NanoString Analysis

**Figure 5:**
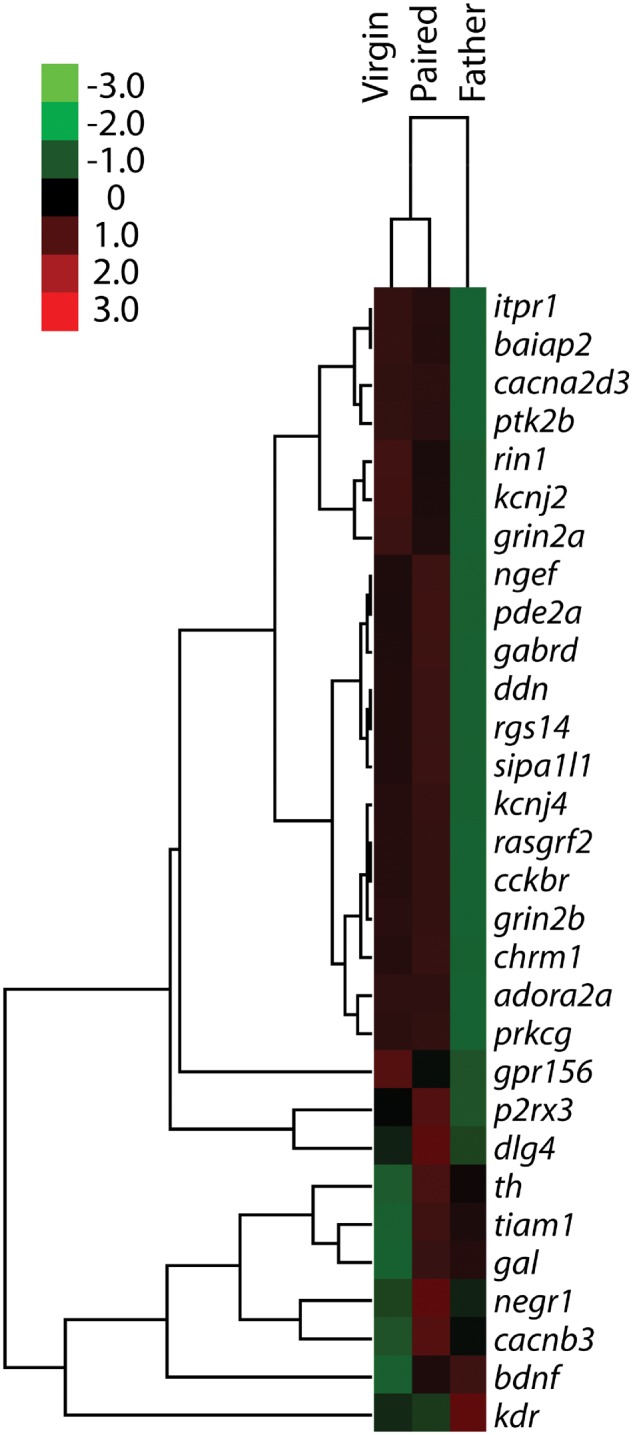
heat map representing the relative expression of individual genes in virgin males, paired males, and males with fathering experience. Gene enrichment is encoded in the heat map ranging from low (green) to high (red). Genes that show similar expression patterns are clustered together, as indicated by the dendrogram to the left of the heat map

**Figure 6:**
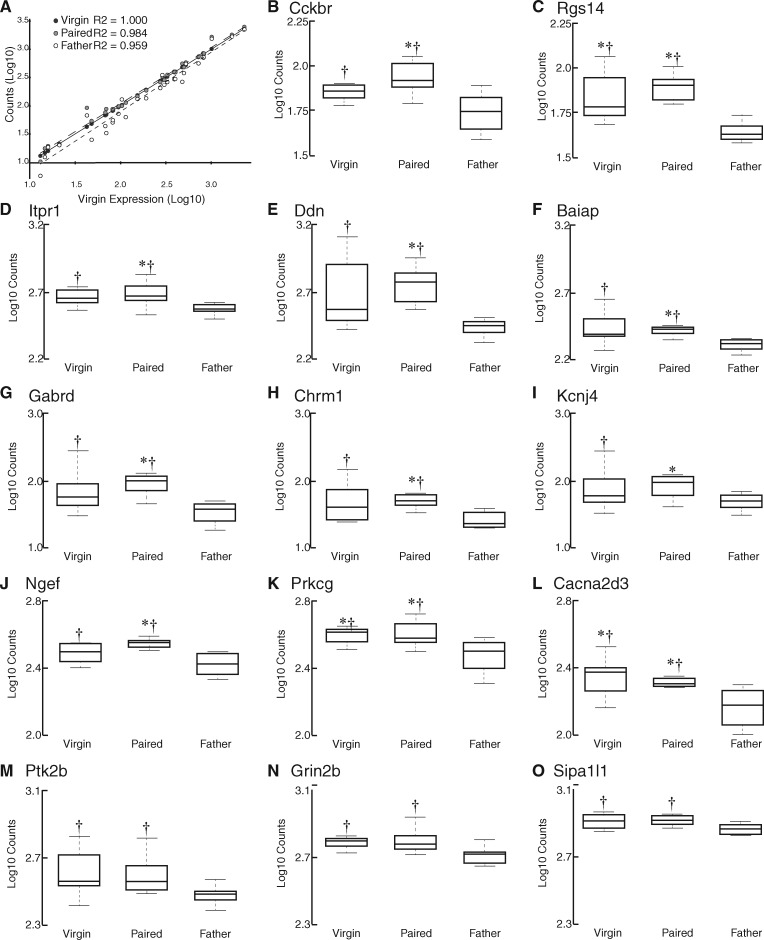
quantitative analysis of gene expression. (**A**) A scatterplot showing the distribution of gene expression in virgin males (black circles), paired males (gray circles), and fathers (white circles). (**B–O**) Box and whisker plots showing the expression of genes in virgins, paired males, and fathers. The whiskers represent × 1.5 the interquartile range. While we quantified the expression of 30 genes using NanoString, here we show the 14 genes that exhibited significantly different levels of expression across groups or exhibited large effect size. In each gene of these genes, expression in fathers was lower than expression in paired males, and in three cases (Rgs14, Prkcg, and Cacna2d3) expression in fathers was also significantly lower than in virgin males. *Significantly differs from fathers (*P* < 0.05). ^†^Large effect size compared with fathers (Cohen’s *d* > 0.8).

A total of 33 genes (30 target genes and 3 housekeeping genes) were selected for quantitative analysis using NanoString. The housekeeping genes (*Gusb*, *Pgk1*, and *Eif4a2*) did not show different levels of expression across conditions, confirming that these genes can serve as a good baseline in prairie voles. A heat map analysis revealed that 23 of our 30 target genes had lower expression levels in fathers than in either virgins or paired males (Fig. 5). Six genes had lower expression levels in virgins, and no gene in any group appeared to show inordinately high levels of expression. A regression analysis revealed similar levels of gene expression across all experimental conditions (Fig. 6A).

Expression data for each individual gene were compared across groups using *t*-tests, which were run and *P*-values were adjusted for multiple comparisons using nSolver software. Of the 30 target genes, 11 genes showed significant differential expression between comparison groups (*P* < 0.05; *Cckbr*, *Rgs14*, *Itpr1*, *Ddn*, *Baiap*, *Gabrd*, *Chrm1*, *Kcnj4*, *Ngef*, *Prkcg*, and *Cacna2d3*; Fig. 6B–O; Table 7). We also calculated the effect sizes using Cohen’s *d*, examining differential expression of each gene across groups (Fig. 6B–O; Table 7). In the V vs P group, we saw a large effect (defined as 0.8 < *d* < 1.2) in Tiam1. In the P vs F group, we saw large effects (0.8 < *d* < 1.2) in *Baiap2*, *Cacna2d3*, *Cckbr*, *Chrm1*, *Ddn*, *Dlg4*, *Gabrd*, *Itpr1*, *Kdr*, *P2rx3*, *Pde2a*, *Ptk2b*, and *Rasgrf2.* In the P vs F group, we also saw very large effects (defined as *d* > 1.2) in *Grin2b*, *Ngef*, *Prkcg*, *Rgs14*, and *Sipa1l1*. In the V vs F group we saw large effects (0.8 < *d* < 1.2) in *Adora2a*, *Cacna2d3*, *Cckbr*, *Chrm1*, *Ddn*, *Gabrd*, *Grin2a*, *Grin2b*, *Itpr1*, *Kcnj4*, *Ngef*, *Prkcg*, *Ptk2b*, and *Sipa1l1*. In the V vs F group we also saw very large effects (*d* > 1.2) in *Baiap2*, *Rgs14*, and *Rin1.*

**Table 7:** *P*-values and Cohen’s *d* values

Gene name	F vs P		F vs V		P vs V	
	*P*-value	Cohen’s *d*	*P*-value	Cohen’s *d*	*P*-value	Cohen’s *d*
*Adora2a*	0.1014	0.5380	0.2444	**0.9243**	0.9891	0.3863
*Baiap2*	**0.0014**	**0.9039**	0.0776	***1.2258***	0.7946	0.3220
*Bdnf*	0.6273	0.2066	0.2702	0.6260	0.4012	0.4194
*Cacna2d3*	**0.0460**	**1.0766**	**0.0326**	**1.1004**	0.9266	0.0239
*Cacnb3*	0.4862	0.4467	0.5735	0.3052	0.2516	0.7520
*Cckbr*	**0.0120**	**0.9881**	0.0767	**0.9884**	0.7745	0.0003
*Chrm1*	**0.0050**	**0.9371**	0.0876	**1.0050**	0.7353	0.0679
*Ddn*	**0.0021**	**1.0688**	0.0829	**1.0685**	0.6383	0.0003
*Dlg4*	0.2146	**0.8759**	0.6877	0.1721	0.2711	0.7037
*Gabrd*	**0.0018**	**0.9480**	0.0803	**0.9667**	0.5384	0.0187
*Gal*	0.6783	0.2902	0.2519	0.4687	0.1944	0.7589
*Gpr156*	0.8590	0.0103	0.7849	0.2844	0.8507	0.2947
*Grin2a*	0.2294	0.6531	0.1292	**0.8749**	0.7381	0.2218
*Grin2b*	0.0778	***1.2351***	0.0877	**1.0075**	0.7291	0.2276
*Itpr1*	**0.0500**	**0.8004**	0.0906	**1.0156**	0.8406	0.2152
*Kcnj2*	0.3466	0.5576	0.1956	0.7754	0.7144	0.2178
*Kcnj4*	**0.0474**	0.6183	0.2362	**0.8591**	0.8533	0.2407
*Kdr*	0.2402	**0.8634**	0.3649	0.6950	0.8613	0.1683
*Negr1*	0.5673	0.3141	0.8722	**0.1085**	0.4501	0.4227
*Ngef*	**0.0061**	**1.1987**	0.1282	**0.9933**	0.5444	0.2054
*P2rx3*	0.0605	**1.1447**	0.3671	0.4699	0.2208	0.6748
*Pde2a*	0.0677	**0.9381**	0.2581	0.7403	0.6827	0.1978
*Prkcg*	**0.0427**	***1.2933***	**0.0390**	**1.1543**	0.8554	0.1389
*Ptk2b*	0.0729	**0.8524**	0.0996	**0.9836**	0.9024	0.1313
*Rasgrf2*	0.1027	**0.9509**	0.1289	0.7855	0.8217	0.1654
*Rgs14*	**0.0002**	***1.5018***	**0.0237**	***1.2370***	0.4867	0.2648
*Rin1*	0.1646	0.7439	0.0696	***1.4011***	0.2535	0.6572
*Sipa1l1*	0.0612	***1.2207***	0.1155	**0.9080**	0.5573	0.3127
*Th*	0.4437	0.3234	0.4343	0.4063	0.1845	0.7297
*Tiam1*	0.7299	0.3216	0.2976	0.5269	0.2510	**0.8484**

*P*-values: bold indicates significant values (*P* ≤ 0.05). Cohen’s *d* values: bold indicates large effect (0.8 < *d* < 1.2). *Bold italics* indicates very large effect (*d* > 1.2).

## Discussion

In this experiment, we compared gene expression in the MPOA of virgin male prairie voles, males that had formed a pair bond, and males with fathering experience. We found that these groups differed in gene expression. Distinct patterns were revealed using a series of analyses, including GO annotation enrichment, KEGG pathways, STRING network analysis, and quantitative assessment using NanoString. Males with fathering experience showed a relative decrease in gene expression compared with virgins and paired males, and many of the genes that exhibited decreased expression were involved in synaptic transmission and plasticity. These results suggest that fathers may exhibit a decreased amount of synaptic plasticity within the MPOA.

The transition to fatherhood is associated with a variety of potential environmental and behavioral changes, such as the presence of infants, changes in energetic requirements and feeding behavior, and stress responsiveness [50–52]. In this experiment, we sought to identify alterations in central nervous system gene expression that are associated with fathering experience. To our knowledge, this is the first time that RNA sequencing has been performed on prairie vole brain tissue. As such, we faced several technical challenges over the course of this study. For instance, while the prairie vole genome has been sequenced [53, 54], its annotation is incomplete, leaving us to rely on the annotated mouse genome (*Mus musculus*) for many of our analyses. In addition, it is important to consider the consequences associated with working in an outbred rodent-like prairie voles. The individual differences associated with an outbred population may have masked additional target genes associated with the onset of paternity. Furthermore, in this study we concentrated on traditional analyses. By examining protein coding transcripts, and restricting our analysis to pathways and genes that were related to the brain and behavior, it is possible that we overlooked some nontraditional candidate genes. Regardless, we still observed significantly altered expression on both the individual gene and system level. These results suggest that paternity engages similar physiological mechanisms across prairie vole males despite genetic diversity.

Biparental care is rare in mammals, but prairie voles are not the only rodents who exhibit this behavior. The males of several species of *Peromyscus*, including *Peromyscus californicus* and *Peromyscus polionotus*, exhibit paternal care, while other species, including *P.maniculatus*, do not. This behavioral distinction allowed Bendesky *et al.* to investigate genetic differences between *P. polionotus* and *P. maniculatus* that are linked to parenting behavior [55]. In a series of experiments, they identified several quantitative trait loci that were linked to specific behaviors of interest, including nest building. Further analysis revealed that the gene for arginine vasopression (AVP) was directly related to nest building, and when AVP was administered intracerebroventricularly there was a significant decrease in the quality of nest building [55]. Unlike, the study by Bendesky *et al.*, we did not find changes implicating AVP. However, there are several differences between the two experiments. In this study, we specifically examined gene expression within one hypothalamic nucleus, the MPOA. Our study was in a different species and used males that had very specific social experiences: virgin males, pair bonded males, and males with fathering experience.

RNA sequencing is a powerful technique that allows us to identify alterations in gene expression that are associated with behavioral and other phenotypic changes [56]. The greatest challenge with this technique, however, is the large amount of data it produces. There is no one agreed upon analysis that most effectively identifies specific genes of interest [57, 58]. Thus, in this study we used several techniques to reveal novel gene targets to further our understanding of paternal behavior. We believe that this is a strength rather than a weakness. The ultimate goal of this experiment was to increase our understanding of the alterations that occur within the MPOA following exposure to different social contexts in male prairie voles. As such, we have identified a set of genes and their associated pathways that we can use to further explore male parenting behavior.

Our quantitative assessment of gene expression in the MPOA revealed an overall decrease in the expression of many genes in fathers relative to both virgins and pair-bonded males. The specific genes of interest that we identified were involved in a range of physiological processes, including metabolism, stress responsiveness, and plasticity. However, most of the genes that showed different patterns of expression between groups, and specifically decreased expression in fathers, were associated with synaptic transmission and dendritic spine motility (Table 8). For example, several genes involved in the production and maintenance of receptors (including *Cckbr*, *Chrm1*, *Gabrd*, *Grin2b*, and *Itpr1*) and ion channels (including *Cacna2d3*, *Kcnj4*, and *P2rx3*) were significantly downregulated. These results suggest that GABA, glutamate, and cholinergic systems are all affected by fathering experience, as are calcium and potassium channels. Other genes that exhibited significant downregulation in fathers were involved with the actin cytoskeleton, dendritic spine motility, and other components of the physical plasticity of dendrites. We emphasize that this is not an exhaustive list of differentially expressed genes; however, these results suggest that synaptic plasticity may be diminished in the MPOA of male prairie voles with fathering experience.

**Table 8:** genes of interest

Gene ID	Gene name	Function	GO Annotations
Baiap2	Brain-specific angiogenesis inhibitor 1-associated protein 2	Insulin receptor tyrisone kinase substrate	Signaling, regulation of biological quality, membrane part, dendrite, dendritic spine, synapse
Cacna2d3	Calcium voltage gated channel, auxiliary subunit alpha 2 delta 3	Voltage gated calcium channel	Ion channel activity
Cckbr	Cholecystokinin B receptor	Multipass transmembrane receptor protein	Signaling, regulation of biological quality
Chrm1	Cholinergic receptor, muscarinic 1	Muscarinic receptor	Signaling, regulation of biological quality, membrane part, dendrite, synapse
Ddn	Dendrin	Plasma membrane surrounding dendritic spine	Membrane part
Gabrd	GABA A receptor, subunit delta	GABA receptor	Signaling, dendrite, synapse, ion channel activity
Grin2b	Glutamate receptor, ionotropic, NMDA 2b	NMDA receptor	Signaling, regulation of biological quality, membrane part, dendrite, dendritic spine, synapse, ion channel activity
Itpr1	Inositol 1,4,5-triphosphate receptor 1	Calcium channel	Signaling, regulation of biological quality, membrane part, dendrite, synapse, ion channel activity
Kcnj4	Potassium voltage gated channel subfamily J member 4	Potassium channels – ion homeostasis	Membrane part, dendrite, synapse, ion channel activity
Ngef	Neuronal guanine nucleotide exchange factor	Dendritic spine morphogenesis	Signaling, membrane part
P2rx3	Purinergic receptor p2x, ligand gated ion channel	ATP receptor	Signaling, regulation of biological quality, membrane part, dendrite, dendritic spine, synapse, ion channel activity
Pde2a	Phosphodiesterase 2a	2nd messenger signaling/dendritic spines	Signaling, regulation of biological quality, membrane part, dendrite
Prkcg	Protein kinase c gamma	Signaling protein	Signaling, membrane part, dendrite, synapse
Ptk2b	Protein tyrosine kinase 2 beta	Ion channel regulation; MapK signaling	Signaling, regulation of biological quality, membrane part, dendrite, dendritic spine, synapse, ion channel activity
Rgs14	Regulator of g-protein signaling 14	Scaffold protein	Signaling, regulation of biological quality, membrane part, dendrite, dendritic spine, synapse
Rin1	Ras and Rab interactor 1	Ras effector	Signaling, regulation of biological quality, membrane part, dendrite
Sipa1l1	Signal induced proliferation associate 1 like 1	Ras effector	Signaling, regulation of biological quality, membrane part, dendrite, dendritic spine, synapse

We were surprised by the lack of differential expression of oxytocin and vasopressin-related genes; however, this finding is not unique within the literature. In a series of experiments, Kenkel *et al.* examined the neuroendocrine correlates of pup exposure in male prairie voles that were virgins or had fathering experience [52, 59]. They saw changes in OT immunoreactivity in PVN/BNST, but there were no changes to OT/AVP in the MPOA. Another study examined OT immunoreactive cells in male prairie voles that were virgins, had established pair bonds, or had fathering experience [60]. They saw an increase in the number of OT immunoreactive cells in the MPOA of paired males and fathers compared with virgin males, but there was a greater increase of OT-ir cells in the PVN of fathers compared with paired and virgin males. It is likely that examination of gene expression in the PVN would show alterations in OT gene expression. In future studies we hope to examine patterns of gene expression in additional brain regions.

Fatherhood also seems to be associated with structural alterations in neural plasticity, as measured by changes in the number and density of dendritic spines. Mice with fathering experience show increased survival of newborn neurons and increased dendritic spine density within the hippocampus [61, 62]. Male marmosets show an increase in dendritic spine density in the prefrontal cortex after fathering experience [63]. However, other studies have shown reductions in the survival of adult-generated neurons in the amygdala of the prairie vole and hippocampus of California mice [64, 65]. The effects of fatherhood clearly vary across brain regions, but we do not yet know what is causing these changes in neural plasticity.

The lower gene expression related to dendritic spines, associated with fatherhood in the present study, is evocative of similar changes seen in a recent study of the MPOA of mother rats [66]. *Rem2*, a gene associated with reduction of dendritic branching but increases in spine density [67, 68] was increased in the MPOA of high licking/grooming rats, but only in lactating mothers (not in virgins). This increase was accompanied by decreased dendritic complexity. *Rem2* is involved with GTPase activity and GTP binding. While we did not see alterations in *Rem2* expression in this study, we found altered expression in several genes that are involved in Ras and Rap1 signaling. Both Ras and Rap1 are GTPases that work in concert to modulate cellular growth and plasticity [69–71]. Ras relays NMDA receptor signaling that drives the delivery of AMPA receptors during LTP, while Rap1 is involved in the NMDA receptor-dependent removal of AMPA receptors during LTD [69, 72]. The altered pattern of expression of these genes in fathers suggests that this extremely salient social experience triggers a molecular cascade that is involved in neuronal plasticity.

The down-regulation of genes associated with dendritic complexity in the present study, as well as the study by Parent *et al.*, is similar to what one would expect in an animal that had experienced high amounts of stress. It is well established that stress, mediated by corticotropin-releasing hormone, results in a loss of dendritic spines [73–77]. Additionally, rat mothers show a decrease in the number and density of dendritic spines in the amygdala and stria terminalis 4 days after birth [78], and an increase in dendritic spine density in the hippocampus during the postpartum period [79]. This suggests that alterations in dendritic spine density in mothers may be both transient and region specific, perhaps linked to the peak in corticosterone that occurs during parturition [80]. More studies must be done to determine if the same holds true for vole fathers.

In many species, the transition to fatherhood is associated with a suite of behavioral and hormonal changes, including those indicative of stress. In California mice (*P.californicus*), fathers exhibit attenuated anxiety-like behavior ∼2 weeks after pups are born [61, 62]. Human males show a peak in cortisol levels during the transition to fatherhood [81]. Prairie vole fathers show increased anxiety-like behavior, and chronic pup exposure (in this case, 20 min of unrelated pup exposure per day for 10 days) results in an increase in basal CORT levels [64]. In an open field test, fathers spent more time in corner squares, and in an elevated plus maze, fathers spent less time in open arms. In forced swim tests, fatherhood decreased the latency to immobility, and increased the number and duration of immobility bouts [64]. In the long-term, fatherhood may be beneficial for male health, but the transition to fatherhood is a tremendously stressful period [82].

In male voles with fathering experience, we also see the upregulation of genes related to protein processing in the endoplasmic reticulum. The endoplasmic reticulum is instrumental in managing the protein folding process, including disposing of misfolded proteins [83]. Homeostatic imbalances, including stress, can alter the functioning of the endoplasmic reticulum, leading to the initiation of the unfolded-protein response, which can in turn lead to apoptosis [84, 85]. This may be one mechanism by which physiological stress can result in homeostatic perturbations [86, 87], including some of the changes that are evident in vole fathers, such as weight loss [50, 59].

While many of the changes we saw in gene expression may be partially attributable to stress, there are likely many other additional factors at play. Fathers in many species show systematic endocrine changes [22]. Environmental factors, including changes in the types and amount of sensory stimulation, or the amount of parental care they received, may play a role as well [88, 89]. Much more work must be done to tease apart these many factors.

In this study, we saw the most varied and interesting differences between the paired males and males with fathering experience. This was surprising, as we expected that the greatest differences would be between the virgin males and fathers. However, examination of the quantitative results begins to clarify these findings (Figs 3 and 6). The expression of genes of interest is slightly elevated in paired animals relative to virgins, but the expression in fathers is decreased relative to virgins. Thus, while the expression levels of some genes do not significantly differ between virgins and paired males, and virgins and fathers, we found significant differences between paired males and fathers. This may suggest that the experience of fathering is functionally distinct from any other type of social interactions that these animals have encountered.

### Conclusions

The purpose of this study was to explore how gene expression changed across the transition to fatherhood, and to identify novel targets to allow for deeper investigation of male parenting behavior. The use of RNA sequencing confirmed that there are differences in gene expression between voles that had different social experiences, including virgin males, males that had formed a pair bond with a female, and males with parenting experience. The genes identified in this study suggest novel processes that are related to paternal behavior and offer new targets for the further exploration of fathering behavior.
